# Impact of Maternal Antibodies on Infectious Bronchitis Virus (IBV) Infection in Primary and Secondary Lymphoid Organs of Chickens

**DOI:** 10.3390/vaccines11071216

**Published:** 2023-07-07

**Authors:** Ishara M. Isham, Mohamed S. H. Hassan, Reham M. Abd-Elsalam, Hiruni A. Ranaweera, Motamed E. Mahmoud, Shahnas M. Najimudeen, Awais Ghaffar, Susan C. Cork, Ashish Gupta, Mohamed Faizal Abdul-Careem

**Affiliations:** 1 Health Research Innovation Center 2C53, Faculty of Veterinary Medicine, University of Calgary, 3330 Hospital Drive NW, Calgary, AB T2N 4N1, Canada; fathimaishara.muhamm@ucalgary.ca (I.M.I.); msh.hassan@ucalgary.ca (M.S.H.H.); reham.abdelsalam1@ucalgary.ca (R.M.A.-E.); hiruni.ranaweera@ucalgary.ca (H.A.R.); motamed.ali@ucalgary.ca (M.E.M.); fathimashahnas.moham@ucalgary.ca (S.M.N.); awais.ghaffar@ucalgary.ca (A.G.); sccork@ucalgary.ca (S.C.C.); ashish.gupta1@ucalgary.ca (A.G.); 2 Department of Poultry Diseases, Faculty of Veterinary Medicine, Assiut University, Assiut 71515, Egypt; 3 Faculty of Veterinary Medicine, Cairo University, Giza 12211, Egypt; 4 Department of Animal Husbandry, Faculty of Veterinary Medicine, Sohag University, Sohag 82524, Egypt

**Keywords:** infectious bronchitis virus, maternal antibody, bursa of Fabricius, thymus, cecal tonsils, spleen

## Abstract

Infectious bronchitis virus (IBV) causes infectious bronchitis disease in chickens. IBV primarily infects the upper respiratory tract and then disseminates to other body systems including gastrointestinal, reproductive, and urinary systems. Unlike original IBV serotypes, the novel IBV variants target lymphoid organs, but information on this is scarce. In this study, we aim to evaluate the impact of the presence of maternal antibodies on IBV infection in primary and secondary lymphoid organs. Maternal antibody free, specific pathogen free (SPF) hens were divided into vaccinated and non-vaccinated groups. The progeny male chicks from these hens were divided into four groups; vaccinated challenged (VC), non-vaccinated challenged (NVC), vaccinated non-challenged (VNC), and non-vaccinated non-challenged (NVNC). The challenge groups were given 1 × 10^6^ embryo infectious dose (EID)_50_ of IBV Delmarva (DMV)/1639 by the oculo-nasal route and non-challenge groups were given saline. The serum anti-IBV antibody titer was significantly higher in challenged groups compared to non-challenged groups. The IBV genome load was significantly lower in the VC group than NVC group in oropharyngeal and cloacal swabs and in bursa of Fabricius (BF) and cecal tonsils (CT). The histopathological lesion scores were significantly lower in VC group than NVC group in BF and CT. These findings suggest that the presence of maternal antibody in chicks could provide some degree of protection against IBV infection in BF and CT.

## 1. Introduction

Infectious bronchitis (IB) is a contagious viral disease caused by a gamma coronavirus known as infectious bronchitis virus (IBV) [1]. IB is one of the most prevalent respiratory diseases in the poultry industry in Canada and elsewhere and it causes significant mortality and morbidity in both broiler and layer chickens, resulting in significant economic impacts to the poultry industry [2,3]. Moreover, in recent years, the Delmarva (DMV)/1639 variant of IBV has been increasingly reported in layer flocks in Eastern Canada [4,5,6]. The DMV/1639 IBV has been linked to cystic oviducts and false layer syndrome outbreaks in layers [7], in addition to a significant drop in egg production (about 30%) when layers were infected at the peak of egg production [8]. As the spread of IBV is one of the major challenges faced by the poultry industry, both live attenuated and killed vaccines developed against common IBV variants, such as Massachusetts (Mass) and Connecticut (Conn), are administered to control IB [9]. As a common practice live-attenuated vaccines are given as spray to one day old chicks at the hatchery before they are transferred to barns, and then a range of live attenuated vaccines are administered along with a killed IB vaccine given pre lay [8]. It is a common practice in the poultry industry to use a combined vaccination regimen with two or more antigenically distinct IB vaccines to provide protection against heterologous IBV challenge [10]. A previous study demonstrated a low nucleotide (77.2%) and amino acid (73.2–75.5%) similarity in the S1 region of the S protein between Canadian IBV DMV/1639 strain and live Mass and Conn vaccine strains [5]. However, another study evaluating the degree of protection against the experimental challenge of IBV DMV/1639 strain in layers after a vaccination schedule of live priming by Mass and Conn types of vaccines as well as boosting with an inactivated vaccine containing Mass and Ark antigens reported that this vaccination routine provided protection against a challenge with DMV/1639 strain in layers [8].

Although IBV primarily infects the upper respiratory tract, it can show tropism to other tissues depending on the strain of the virus. Although the IBV DMV/1639 variant was first reported as a nephropathogenic strain [11], in recent years, studies have demonstrated that it can infect the reproductive tract, cecal tonsils (CT), and kidney [7,8]. There is no extensive literature on the ability of IBV to infect immune organs, but a few studies have reported the detection of IBV genome in lymphoid organs, such as the bursa of Fabricius (BF) [9,12,13], thymus [9,12], CT [7,8,14,15], and spleen [12]. Furthermore, some studies have also demonstrated the presence of IBV-induced microscopic lesions in lymphoid organs, such as BF [9,12,13] and spleen [12]. The lymphoid organs play a pivotal role in mounting an immune response against infections, and in chickens, the thymus and BF are primary lymphoid organs that produce T cells and B cells, respectively [16,17]. Meanwhile, secondary lymphoid organs such as the CT, spleen, and harderian gland (HG) and mucosa associated lymphoid tissues (MALT) such as bronchial associated lymphoid tissues (BALT) and gut-associated lymphoid tissues (GALT) allow the accumulation and maturation of T and B cells at the later stages of life in the chicken [16,17].

Maternal antibodies are transferred from vaccinated hens to their progeny via the yolk and can be detected in the serum and respiratory mucus of newly hatched chicks [18,19]. Moreover, the concentration of maternal antibody in the serum of chicks steadily declines with time and is almost nonexistent by 4 to 5 weeks [18,19,20]. There is limited literature on the role of protection provided by maternal antibodies against IBV infection in young chicks [18,19,21]. One such study showed that the presence of maternal antibodies in serum provided protection against challenge from IBV as chicks with maternal antibodies had a lower percentage of mortality [18]. Another study by Mondal et al. in 2001 reported that maternal antibodies in the respiratory mucus of chicks were responsible for protection against challenge by IBV as protection to challenge by IBV dropped significantly by 1 week along with the drop in maternal antibodies in the respiratory mucus [19].

There is a scarcity of knowledge regarding the role of maternal antibodies against IBV infection in the chickens, and there are little to no studies on IBV infection in lymphoid organs. Furthermore, the limited literature on the role of maternal antibodies against IBV infection in hatched chicks is focused on determining if the presence of the maternal antibodies provide protection against IBV infection in the respiratory tract [18,19,20]. In addition, there is no literature on the impact of the presence of maternal antibodies with respect to IBV infection in lymphoid organs. Since the lymphoid organs play a key role in controlling IBV infection in the chicken, in this study, our objective was to investigate if the presence of maternal antibodies in hatched chicks provides protection against IBV infection of primary (BF and thymus) and secondary (CT and spleen) lymphoid organs.

## 2. Materials and Methods

### 2.1. Virus and the Animals

The Canadian IBV DMV/1639 strain used in this study, designated as IBV/Ck/Can/17-036989, was isolated, propagated, and titrated as previously described [5,7]. Ninety-one (77 females and 14 males), one-day old White Leghorn SPF chickens (*Gallus gallus domesticus*) were purchased from the Canadian Food Inspection Agency (CFIA), Ottawa, Ontario, Canada and were raised for 30 weeks of age until egg production in the Veterinary Science Research Station (VSRS) at the Spy Hill campus, University of Calgary. The young chicks for the experiment were obtained by hatching the eggs collected from these White Leghorn SPF hens by incubating at Health Research Innovation Center (HRIC) 2C53, Foothills campus, University of Calgary. The hatched male chicks were housed in the VSRS at the Spy Hill campus, University of Calgary. The ethical approval for this proposed work was obtained from the Health Science Animal Care Committee (HSACC) of the University of Calgary (Protocol number: AC19-0011). All the experiments were conducted in negative pressure rooms.

### 2.2. Experimental Design

**Figure 1:**
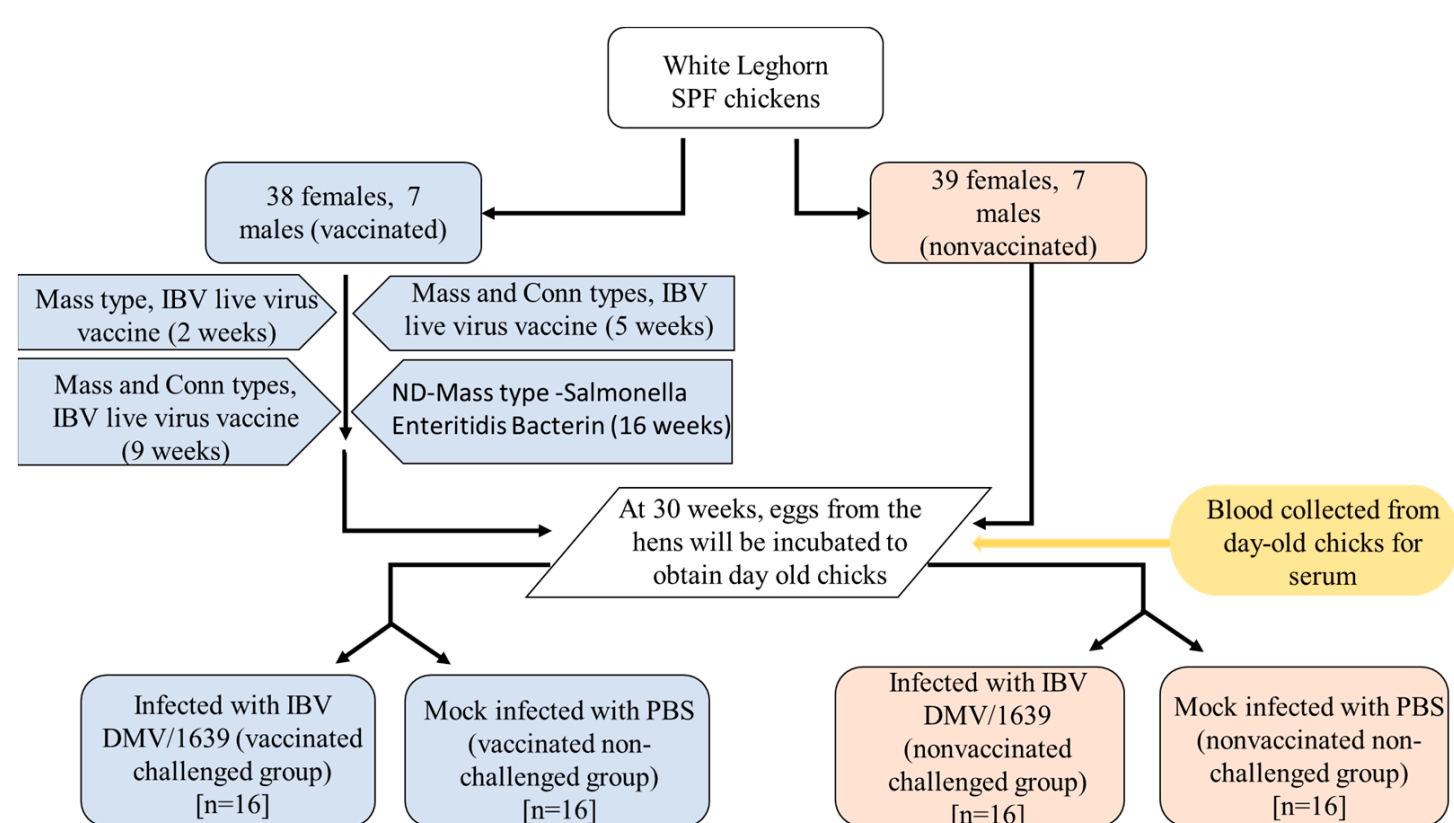
Summary of the experimental design used in this study.

The White Leghorn SPF chickens (*n* = 91) were divided into two groups, named as vaccinated (38 females and 7 males) and non-vaccinated controls (39 females and 7 males). The birds in the vaccinated group were vaccinated with live attenuated Mass vaccine (Bronchitis Vaccine, Zoetis Inc., Kalamazoo, Michigan, United States of America, or USA) at 2 weeks of age and Mass and Conn IB vaccine (Bronchitis Vaccine, Zoetis Inc., Kalamazoo, MI, USA) at 5 and 9 weeks of age. Finally, at 16 weeks of age, Newcastle Disease (ND)-Bronchitis Vaccine-Salmonella Enteritidis Bacterin, Mass type, killed virus vaccine (Bronchitis Vaccine, Zoetis Inc., Kalamazoo, MI, USA) was given to the vaccinated pullets. At 30 weeks of age, eggs were collected from vaccinated and non-vaccinated hens, incubated, and hatched to obtain progenies. Day-old male chicks were randomly divided into four groups (*n* = 16), named as vaccinated challenged (VC), non-vaccinated challenged (NVC), vaccinated non-challenged (VNC), and non-vaccinated non-challenged (NVNC). Serum samples were collected from all groups at the day of hatching before the viral challenge. The VC and NVC groups were challenged with 100 μL of IBV DMV/1639 strain at a dose of 1 × 10^6^ embryo infectious dose (EID)_50_ by the oculo-nasal route. The VNC and NVNC groups were mock challenged with 100 μL of phosphate buffered saline (PBS) (Figure 1). At 3 and 7 days post-infected (dpi), under isoflurane anesthesia followed by euthanasia, oropharyngeal (OP) and cloacal (CL) swabs were collected from the birds from each group and were stored in 1 mL aliquots of PBS. In addition, 1 mL of blood was collected, and serum was separated and stored at −20 °C until further processing. The trachea, lung, BF, thymus, CT, spleen, duodenum, pancreas, and kidney tissue samples were collected in RNA Save^®^ (Biological Industries, Beit Haemek, Israel) for extraction of ribonucleic acid (RNA). In addition, samples of all the collected tissues were fixed in 10% neutral buffered formalin (VWR International, Edmonton, AB, Canada) for histopathology evaluation and immunohistochemistry assay. Swabs and tissues collected for RNA extraction were stored at −80 °C until further processing, and tissue samples in 10% neutral buffered formalin were stored at room temperature.

### 2.3. Techniques

#### 2.3.1. Enzyme-Linked Immunosorbent Assay (ELISA)

A commercial ELISA kit (IDEXX Laboratories, Inc., Westbrook, ME, USA) was used to measure the titer of maternal antibodies against IBV in serum at a serum dilution of 1:500 according to the manufacturer’s instructions. The antibody titers were calculated with the formula provided by IDEXX, where sample to positive ratio was calculated and titers above 396 (cut-off) were considered positive.

#### 2.3.2. RNA Extraction and Complimentary Deoxy Ribonucleic Acid (cDNA) Synthesis

The OP and CL swab samples were vortexed and 250 μL of the sample was used to extract the RNA using Trizol LS^®^ reagent (Invitrogen Canada Inc., Burlington, ON, Canada) and total RNA was extracted from tissue samples using Trizol reagent (Invitrogen Canada Inc., Burlington, ON, Canada) according to manufacturer’s guidelines. Following this, the amount of extracted RNA was quantified using a Nanodrop 1000 spectrophotometer (Thermo Scientific, Wilmington, DE, USA.) at 260 nm wavelength. Finally, 1000 ng of RNA from swab samples and 2000 ng of RNA from tissue samples were used to synthesize cDNA using RT random primers high-capacity cDNA reverse transcriptase kit (Invitrogen Life Technologies, Carlsbad, CA, USA) according to the manufacturer’s instructions.

#### 2.3.3. IBV Genome Load Quantification by Quantitative Polymerase Reaction (qPCR) assay

The qPCR assay was performed using Fast SYBR^®^ Green Master Mix (Quntabio^®^, Beverly, MA, USA) with a final reaction volume of 20 µL using cDNA as a template. Each reaction volume consisted of 10 µL of SYBR Green master mix, 100 ng of respective cDNA sample, 0.5 µL of forward primer, 0.5 µL of reverse primer, and molecular biology grade water. All these qPCR assays were carried out using a CFX 96-c1000 Thermocycler (Bio-Rad Laboratories, Mississauga, ON, Canada) and the thermal cycling conditions were initial denaturation at 95 °C for 20 s, followed by 40 cycles of amplification at 95 °C for 3 s, and annealing at 60 °C for 30 s. The primers targeted the conserved IBV nucleocapsid (N) gene where the forward primer and reverse primer sequences were 5′GACGGAGGACCTGATGGTAA-3′ and 5′CCCTTCTTCTGCTGATCCTG-3′, respectively [22].

#### 2.3.4. Immunohistochemistry Technique

All the collected tissue samples were examined using immunohistochemistry technique to identify the presence and localization of the IBV nucleoprotein. Immunohistochemistry was performed following the methods described in the past literature [23]. Initially, the paraffin sections on positively charged slides (VWR International, Radnor, PA, USA) were deparaffinized in two changes of xylene, followed by rehydration in serial concentrations of alcohol in descending order. Then, the tissue specimens were incubated with 3% H_2_O_2_ solution in methanol for 10 min at room temperature to block the endogenous peroxidase activity. Subsequently, the tissue sections were microwaved at 850 W for 15 min with a 10 mM citrate buffer at pH 6.0 to unmask the viral epitopes. The tissue sections were then incubated overnight at 4 °C in a humidified chamber with mouse primary anti-IBV nucleoprotein antibody (Novus Biological, Bio-Techne, Toronto, ON, Canada) diluted 1:400 in PBS. Following this, the tissue sections were incubated with goat anti-mouse IgG (H + L) secondary antibody (Vector Laboratories Inc., Newark, CA, USA), for 30 min at room temperature. Then, ABC peroxidase kit and 3,3’-diaminobenzidine (DAB) substrate solution (Vector Laboratories, Newark, CA, USA) were used according to the manufacturer’s instructions to detect the antibody binding. All the above incubation steps were followed by 5 min of washing with Tris-buffered saline (TBS). Henceforth the slides were counterstained with haematoxylin (Vector Laboratories, Newark, CA, USA) for 8 min, and bluing was achieved by running under tap water for 30 min. The dehydration and cleaning were conducted in an ascending series of alcohol and xylene, respectively. Finally, the tissue sections were cover-slipped using a mounting solution (Vector Laboratories, Newark, CA, USA).

#### 2.3.5. Histopathology

The tissues fixed in 10% neutral buffered formalin were submitted to the Diagnostic Services Unit (DSU) at the University of Calgary, Faculty of Veterinary Medicine for obtaining paraffin embedded tissue blocks and hematoxylin-eosin (H&E) stained and unstained tissue sections on positively-charged slides (VWR International, Radnor, PA, USA). The stained tissue sections were examined under the microscope (Olympus BX51, Center Valley, PA, USA) and scored following the methods previously described with some alteration [24]. There were several criteria for scoring each organ (Table 1), and the scoring system was performed as follows: normal (0), mild (1), moderate (2), and severe (3) for each criterion, and then the total score was calculated for each organ per group.

### 2.4. Statistical Analyses

The mean IBV antibody titer in the serum of pre-challenged birds were compared using unpaired t test while mean antibody titers in post-challenged birds at 3 and 7 dpi were compared using one-way ANOVA followed by Tukey’s multiple comparisons test. IBV genome loads in OP swabs, CL swabs, and tissues were compared using one-way ANOVA followed by Tukey’s multiple comparisons test. The significant differences of histopathological lesion scores between the four groups were analyzed using two-way ANOVA followed by Tukey’s multiple comparison test. The statistical significance between the groups were denoted with different number of asterisks in the graphs, where *, **, ***, and **** implies significance when *p* < 0.05, *p* < 0.01, *p* < 0.001, and *p* < 0.0001, respectively. All the statistical analyses were performed with GraphPad Prism 9.2.0 Software (GraphPad Software, San Diego, CA, USA), and this software was also used to generate graphs.

## 3. Results

### 3.1. Serology

**Figure 2:**
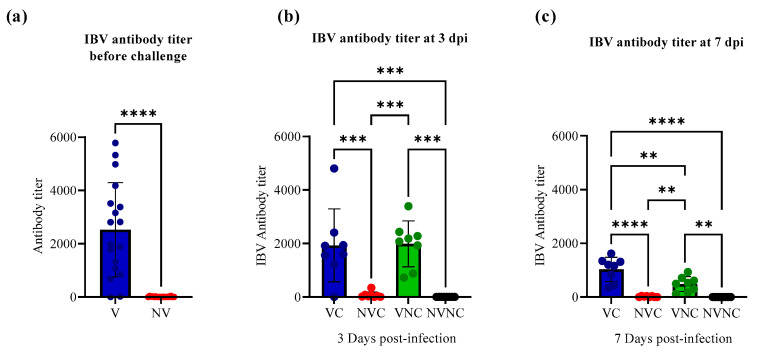
IBV antibody titers in serum of (**a**) pre-challenge birds (*n* = 16/group) at the day of hatching and in serum of post-challenge birds (*n* = 8/group) at (**b**) 3 and (**c**) 7 dpi. Mean antibody titers of pre-challenged birds were compared using unpaired t test. Mean antibody titers in post-challenged birds at 3 and 7 dpi were compared using one-way ANOVA followed by Tukey’s multiple comparisons test. The error bars represent the standard deviation (SD). Statistical significance: ** *p* < 0.01, *** *p* < 0.001, **** *p* < 0.0001.

The antibody titer against IBV in the progeny chicks from vaccinated hens (V) (mean antibody titer = 2529.71) was significantly higher than that in progeny chicks from non-vaccinated hens (NV) (mean antibody titer = 0) (*p* < 0.0001; Figure 2a) before challenge. Following challenge at 3 dpi, a significantly higher level of antibody titer was measured in the VC (mean antibody titer = 1928.67) and VNC (mean antibody titer = 1986.53) groups compared to NVC and NVNC groups, which had no detectable antibodies (*p* < 0.001; Figure 2b). At 7 dpi, the VC (mean antibody titer = 1067.31) and VNC (mean antibody titer = 483.69) groups had a significantly higher level of antibody titer compared to NVC and NVNC groups, which had no detectable antibodies (*p* < 0.01; Figure 2c).

### 3.2. IBV Shedding

**Figure 3:**
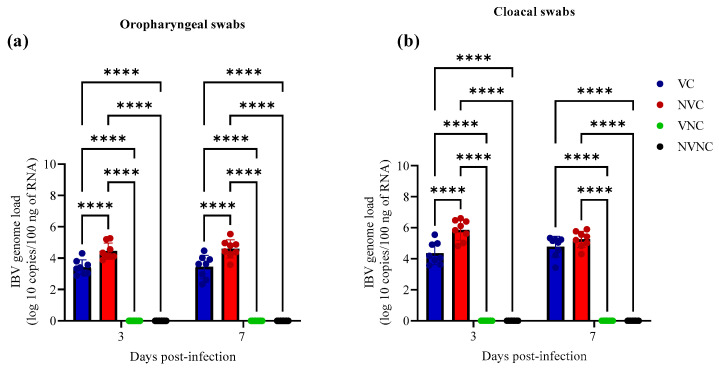
IBV genome loads in (**a**) OP and (**b**) CL swabs at 3 and 7 dpi following challenge with the Canadian DMV/1639 IBV strain (IBV/Ck/Can/17–036989). The average starting IBV genome load was quantified per 100 ng of the extracted RNA and comparisons between groups was performed using one-way ANOVA followed by Tukey’s multiple comparisons test, and the error bars represent the SD. Statistical significance: **** *p* < 0.0001.

IBV genome was not detected in OP and CL swabs of VNC and NVNC groups at 3 and 7 dpi, indicating no other source of IBV challenge (Figure 3). Among challenged groups, the IBV genome load of VC group in OP swabs was significantly lower compared to the NVC group at 3 and 7 dpi (*p* < 0.0001; Figure 3a). In CL swabs, the VC group demonstrated significantly lower IBV genome load compared to NVC group only at 3 dpi (*p* < 0.0001; Figure 3b).

### 3.3. IBV Genome Loads in Tissues

**Figure 4:**
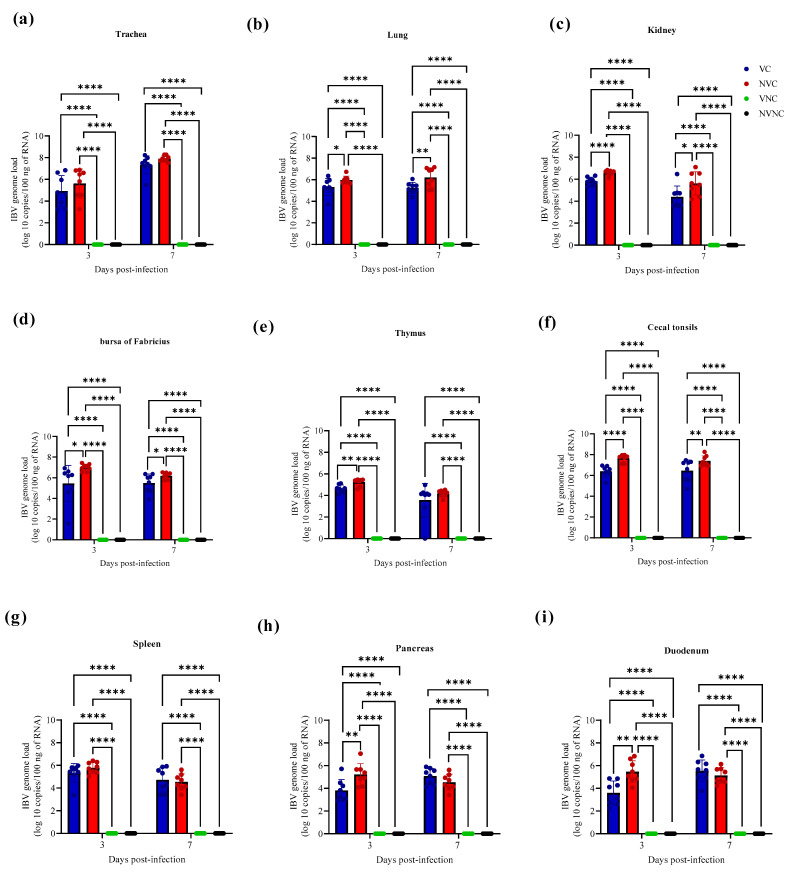
IBV genome loads in (**a**) trachea, (**b**) lung, (**c**) kidney, (**d**) bursa of Fabricius, (**e**) thymus, (**f)** cecal tonsils, (**g**) spleen, (**h**) pancreas, and (**i**) duodenum at 3 and 7 dpi following challenge with the Canadian DMV/1639 IBV strain. The mean IBV genome load was quantified per 100 ng of the extracted RNA and comparisons between groups were determined using one-way ANOVA followed by Tukey’s multiple comparisons test, and the error bars represent the SD. Statistical significance: * *p* < 0.05, ** *p* < 0.01, **** *p* < 0.0001.

The IBV genome was not detected with qPCR assay in all target organs of the VNC and NVNC groups at both 3 and 7 dpi, thus affirming no other source of IBV challenge (Figure 4). The VC group had significantly lower IBV genome loads compared to the NVC group in lung, kidney, BF, and CT at both 3 and 7 dpi (*p* < 0.05; Figure 4b–d,f). In thymus, pancreas, and duodenum, a significantly lower IBV genome loads were observed in the VC group compared to the NVC group only at 3 dpi (*p* < 0.01; Figure 4e,h,i).

### 3.4. IBV Nuclear Antigen in Tissues

**Figure 5:**
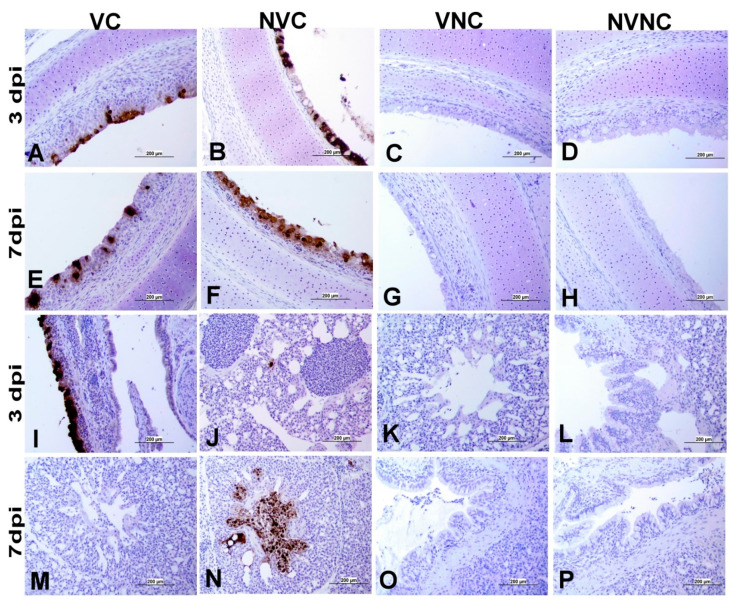
Immunohistochemical analysis of IBV nucleoprotein in trachea and lung at 3 and 7 dpi. IBV nucleoprotein is expressed as brown intra-cytoplasmic staining in (**A**–**H**) trachea and (**I**–**P**) lung.

**Figure 6:**
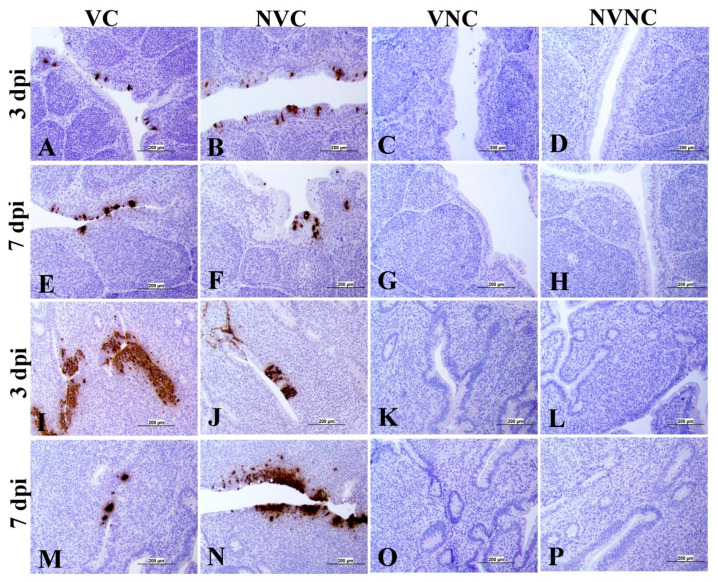
Immunohistochemical analysis of IBV nucleoprotein in bursa of Fabricius (BF) and cecal tonsils (CT) at 3 and 7 dpi. IBV nucleoprotein is expressed as brown intra-cytoplasmic staining in (**A**–**H**) BF and (**I**–**P**) CT.

**Figure 7:**
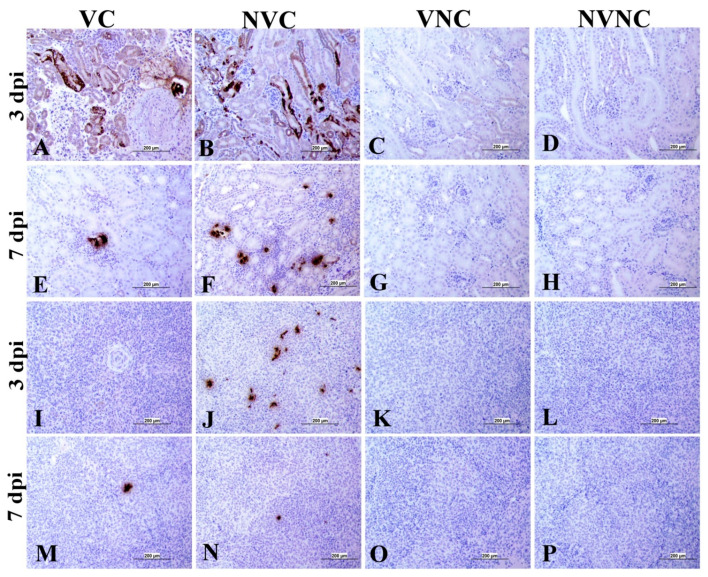
Immunohistochemical analysis of IBV nucleoprotein in kidney and spleen at 3 and 7 dpi. IBV nucleoprotein is expressed as brown intra-cytoplasmic staining in (**A**–**H**) kidney and (**I**–**P**) spleen.

**Figure 8:**
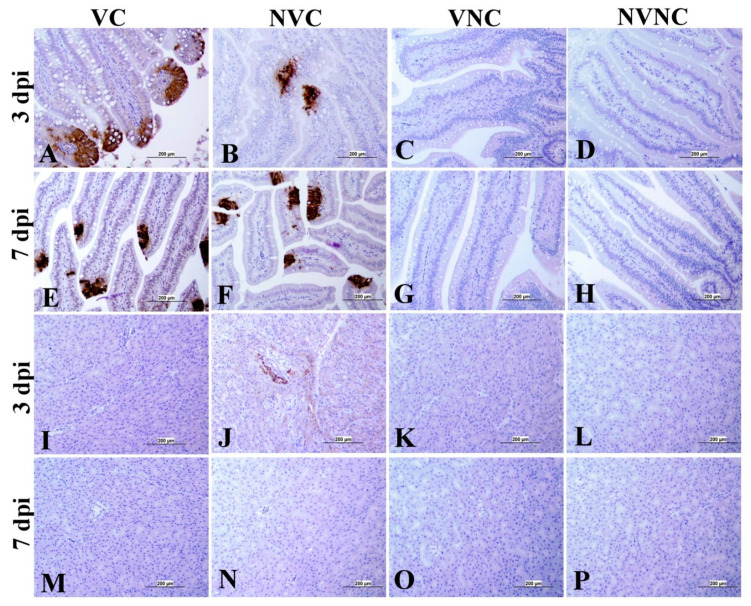
Immunohistochemical analysis of IBV nucleoprotein in duodenum and pancreas at 3 and 7 dpi. IBV nucleoprotein is expressed as brown intra-cytoplasmic staining in (**A**–**H**) duodenum and (**I**–**P**) pancreas.

IBV immune-positive staining was not observed in the non-challenged (VNC and NVNC) groups at 3 and 7 dpi in all target organs (Table 2 and Table 3 and Figure 5, Figure 6, Figure 7 and Figure 8). In contrast, the VC and the NVC groups at 3 and 7 dpi revealed immune-positive staining of IBV nucleoprotein in different tissues. Table 2 and Table 3 summarize the number of birds expressing IBV immune-positive staining in different tissues at 3 and 7 dpi, respectively. Further IBV nucleoprotein expression in different tissues is demonstrated in Figure 5, Figure 6, Figure 7 and Figure 8. The IBV nucleoprotein positive cells exhibited intra-cytoplasmic, brown fine to coarse crumbs, and these cells were predominantly located in mucosal epithelium, renal tubular epithelium, pancreatic acinar cells, and macrophages.

### 3.5. Histopathology

**Figure 9:**
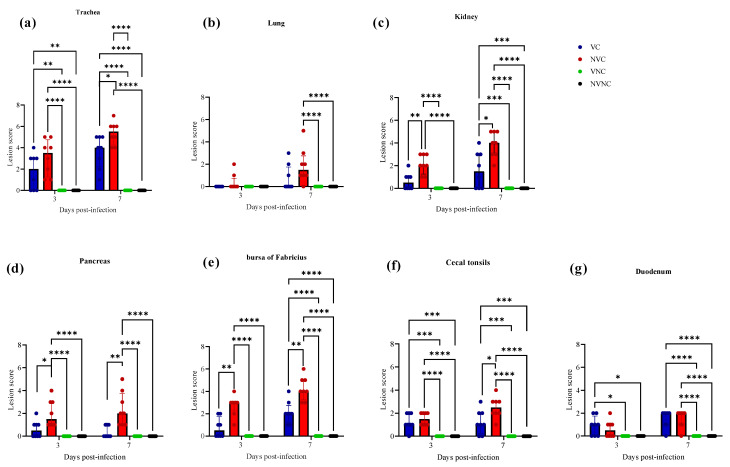
Lesion scores in (**a**) trachea, (**b**) lung, (**c**) kidney, (**d**) pancreas, (**e**) bursa of Fabricius, (**f**) cecal tonsils, and (**g**) duodenum at 3 and 7 dpi following challenge with the Canadian DMV/1639 IBV strain. Error bars represent values expressed as median with interquartile range and were analyzed using two-way ANOVA followed by Tukeys multiple comparisons and the Mann–Whitney test to compare the VC and NVC groups. Significance: * *p* < 0.05, ** *p* < 0.01, *** *p* < 0.001, **** *p* < 0.0001.

**Figure 10:**
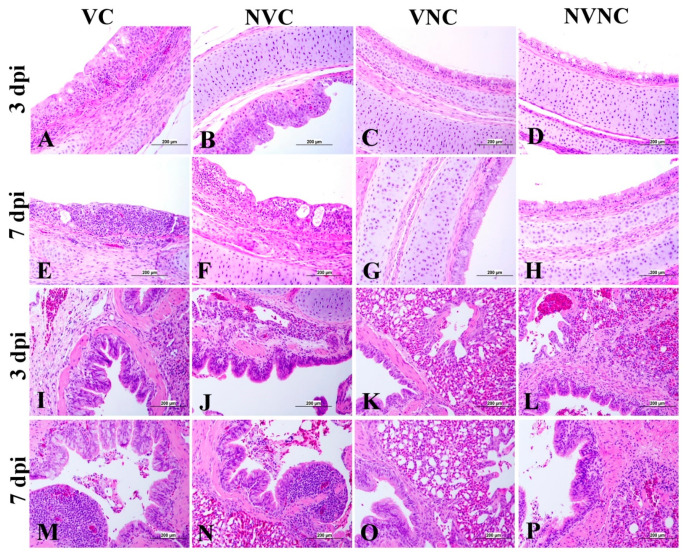
Photomicrographs of trachea and lung representing the four experimental groups. (**C**,**D**,**G**,**H**) Trachea shows normal histological pictures. (**A**,**B**) Trachea shows epithelial lining degeneration and necrosis, and mild mucosal and submucosal mononuclear cell infiltration. (**E**,**F**) Trachea shows marked degeneration and necrosis of epithelial lining with ballooning of mucus cells and severe mucosal and submucosal mononuclear inflammatory cell aggregation. (**K**,**L**,**O**,**P**) Lung shows normal histological architecture. (**I**,**J**) Lung shows mild peribronchial mononuclear inflammatory cell infiltration with degenerative changes in their epithelium lining. (**M**,**N**) Lung shows moderate bronchitis.

No histopathological lesions were detected in thymus and spleen of any experimental groups, and no histopathological lesions were detected in the NVNC and VNC groups in any target organs (Figure 9). There was a significant drop in tracheal lesion score in VC group compared to the NVC group at 7 dpi (*p* < 0.05; Figure 9a). The NVC group revealed loss of cilia, degeneration, and necrosis of some epithelial lining, depletion of mucus glands, and mild mucosal and submucosal mononuclear cell infiltration at 3 dpi. However, these lesions were more severe at 7 dpi. In addition to that, some areas of trachea showed hyperplasia of epithelial and mucus cells with focal areas of mucosal loss (Figure 10). In contrast, the VC group showed less severe tracheal lesions in the form of loss of cilia, mild to moderate epithelial cell degeneration, and necrosis (Figure 10).

There was not any significant difference between the NVC and VC groups in lung score at both 3 and 7 dpi (Figure 9b). The lesions in the lung were minimal in both the VC and NVC groups at 3 dpi while mild primary and secondary bronchitis was observed in both groups at 7 dpi (Figure 10). Moreover, parabronchitis was recorded in one bird of the NVC group.

**Figure 11:**
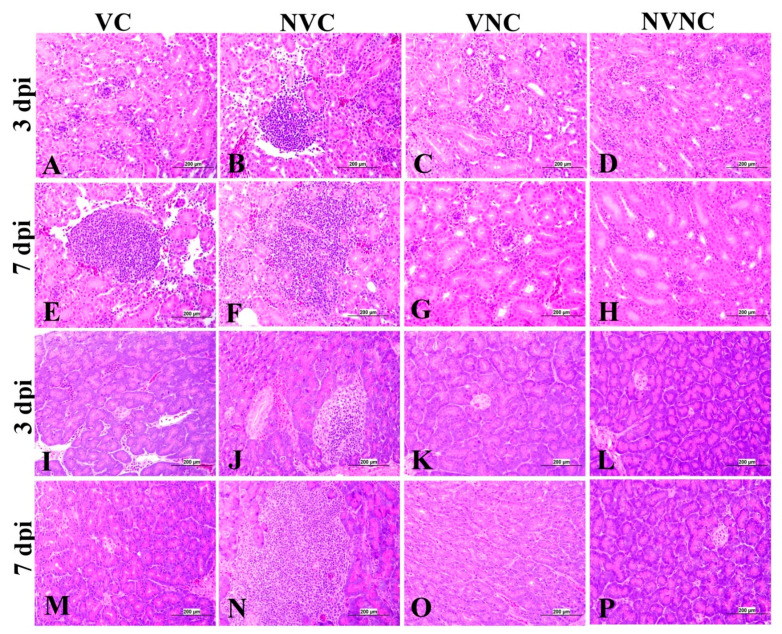
Photomicrographs of kidney and pancreas representing the four experimental groups. (**C**,**D**,**G**,**H**) Kidney shows normal histological structure. (**A**) Kidney shows mild vacuolar degeneration of epithelium lining renal tubules. (**B**,**E**) Kidney shows focal interstitial lumphoplasmocytic nephritis. (**F**) Kidney shows diffuse lymphoplasmocytic interstitial nephritis. (**K**,**L**,**M**,**O**,**P**) Pancreas shows normal histological picture. (**I**) Pancreas shows mild lymphocytic infiltration with degeneration and necrosis of acinar cells. (**J**) Pancreas shows focal lymphocytic infiltration, degeneration, and necrosis of acinar cells and dilation of the intra lobular duct wall with periductular heterophilic aggregation. (**N**) Pancreas shows lymphocytic infiltration.

The VC group showed a significant decrease in lesion scores at 3 and 7 dpi compared to the NVC group in kidney (*p* < 0.05; Figure 9c). The kidney lesions were mild tubular necrosis with focal lymphoplasmocytic aggregations in the interstitial tissues at 3 and 7 dpi in the VC group (Figure 11). One bird of the VC group exhibited severe lymphoplasmocytic interstitial nephritis, whilst the NVC group revealed severe tubular necrosis with cellular cast, focal to diffuse lymphoplasmocytic infiltration in the interstitial tissues, and urates deposition in ureter and some renal tubules (Figure 11).

The pancreatic lesions were more severe and had a significant increase in the NVC group at both 3 and 7 dpi compared to the VC group (*p* < 0.05; Figure 9d). The NVC group showed multifocal to diffuse areas of lymphocytic infiltration with degeneration and necrosis of acinar cells. Infiltration of intra lobular duct walls with heterophils, lymphocytes, and macrophages were also seen (Figure 11). On the other hand, the VC group revealed minute lymphocyte aggregations in the acinar tissue (Figure 11).

**Figure 12:**
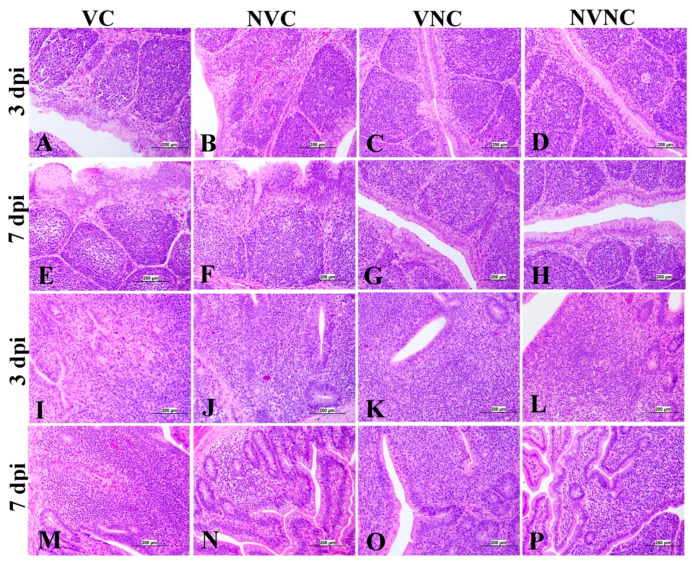
Photomicrographs of bursa of Fabricius (BF) and cecal tonsils (CT). (**C**,**D**,**G**,**H**) BF shows normal histological structure. (**A**) BF shows mild lining epithelial hyperplasia with degeneration and necrosis of the few cells. (**B**) BF shows degeneration and necrosis of its epithelium lining with sub epithelium and interfollicular mononuclear cell and heterophils aggregation. (**E**) BF shows multifocal areas of epithelial cell hyperplasia. (**F**) BF shows severe hyperplasia of lining epithelium with squamous cell metaplasia with apoptosis and necrosis of some of them. (**K**,**L**,**O**,**P**) CT shows normal histological findings. (**I**) CT shows severe lymphoepithelial necrosis and sub-epithelial inflammatory cells aggregation mainly with lymphocytes and macrophages (**J**) CT shows moderate lymphoepithelial necrosis with lymphoidal apoptosis in the interfollicular area. (**M**) CT shows severe lymphoepithelial necrosis with sub-epithelial heterophils, lymphocytes, and macrophages aggregation. (**N**) CT shows vacuolar degeneration, and necrosis of lymphoepithelium with lymphoidal depletion in interfollicular tissue.

The BF of the VC group showed mild to moderate lesions at 3 and 7 dpi, respectively, and a significant lesion score reduction was also detected at 3 and 7 dpi compared to the NVC group (*p* < 0.01; Figure 9e). Mild to moderate epithelial cell hyperplasia and stratified squamous cell metaplasia, degeneration, necrosis of few cells, and mild lymphoid depletion were observed in the VC group (Figure 12), while the NVC group revealed severe epithelial cell hyperplasia, stratified squamous cell metaplasia, degeneration, and necrosis of hyperplastic cells. Expansion of subepithelial and interfollicular tissue with heterophils, macrophages and lymphocytes were also recorded at 3 dpi. Moreover, moderate lymphoid depletion with the accumulation of foamy macrophages in the interfollicular tissue was observed at 7 dpi (Figure 12).

Regarding CT, there was no significant difference in lesion scores at 3 dpi between the VC and NVC groups, whilst a significant difference in lesion scores (*p* < 0.05) was recorded at 7 dpi between these groups (Figure 9f). The NVC group revealed more severe lesions at 7 dpi in the form of lymphoepithelial necrosis with sub-epithelial heterophils, lymphocytes, and macrophages aggregation (Figure 12).

Concerning the duodenum, there was not any significant difference in lesion scores between the VC and NVC groups at 3 and 7 dpi (Figure 9g). The duodenum lesions were mild in the form of mild epithelial degeneration and necrosis, and mild lymphoid infiltration in the lamina propria.

## 4. Discussion

IBV is a highly contagious virus that causes significant economic losses in the poultry industry. Hence understanding host immunity and protection against IBV infection is essential. The role of maternal antibodies against IBV infection in chicken has not been extensively studied in the past. Although a few studies have reported that the presence of maternal antibodies against IBV in hatched chicks provides protection to challenge by IBV during the first week post-hatching, most of these studies were centered on determining if the presence of maternal antibodies provides protection against IBV infection in the respiratory tract [18,19,20]. The impact of the presence of maternal antibodies with regards to IBV infection in lymphoid organs has not been previously studied. Since the lymphoid organs play a prominent role in controlling IBV infection in chicken, in this study, our aim was to determine if the presence of maternal antibodies in hatched chicks provides protection against IBV infection in primary (BF and thymus) and secondary (CT and spleen) lymphoid organs. In the current study, one-day old male chicks obtained from vaccinated and non-vaccinated hens were challenged with Canadian DMV/1639 IBV strain. We have a few distinct findings in this study. First, we observed that the anti-IBV antibody titer in serum of chicks of vaccinated hens were significantly higher compared to chicks of non-vaccinated hens, and, consequently, the viral shedding in OP and CL swabs was significantly lower in chicks of vaccinated hens compared to chicks of non-vaccinated hens. In addition, we found that the IBV genome loads, and histopathological lesions were significantly lower in chicks of VC group compared to chicks from the NVC group in BF and CT.

In the current study, we observed a gradual decline in the serum antibody titer from the day of hatching to 7 days of age in chicks from the VC and VNC groups. This observation is in agreement with the past literature, where a similar pattern of decrease in serum antibody titer has been reported [18,19,20]. Previous studies further stated that the drop in maternal antibody titer is due to the half-life of these antibodies in the young chicks [18,19,20]. Although a gradual drop in antibody titer was observed in the current study, this decrease in serum antibody titer in the VC group was not significant at 0, 3, and 7 dpi. These findings agree with previous studies, which demonstrated that there were no significant changes in the maternal antibody titer in serum until 17 to 35 days of age in young chicks challenged with IBV [18,19]. However, in the chicks from the VNC group, we observed a significant decrease in maternal antibody titer between 0 and 7 dpi and 3 and 7 dpi. These findings are in agreement with maternal antibody decay data of non-challenged chicks observed in previous studies, which report the half-life of these antibodies to be about 4 to 6 days [18,20,25].

In the current study, we detected a significantly higher level of antibody titer in chicks from vaccinated hens (the VC and VNC groups) compared to chicks from non-vaccinated hens (the NVC and NVNC groups) at 3 and 7 dpi, and, correspondingly, the IBV genome load in OP swabs of chicks from the VC group was significantly lower compared to chicks from the NVC group at 3 and 7 dpi. In addition, the IBV shedding observed in CL swabs was significantly lower in the VC group compared to the NVC group at 3 dpi. All these data suggest that the presence of maternal antibodies in serum could provide some degree of protection against IBV infection and replication in young chicks. The study by Mockett et al. in 1987, where eggs from vaccinated hens were hatched, and 1-day-old and one-week-old chicks were challenged with IBV strain VF69-149 (Mass serotype) also reported that the presence of maternal antibodies in serum provided protection against challenge from IBV, as chicks with maternal antibody had a lower percentage of mortality [18]. The past literature further stows that the protection from maternal antibodies is transient, as the percentage of mortality was not lower in older chicks that were 2 to 6 weeks of age [18].

IBV viral genome was detected in all of the tested organs (trachea, lung, kidney, pancreas, duodenum, BF, thymus, CT, and spleen) of IBV challenged birds. A few studies have previously reported the presence of IBV genome in the primary lymphoid organs BF [9,12,13] and thymus [9,12], and in secondary lymphoid organs such as CT [7,8,14,15] and spleen [12], and these results are consistent with the findings in our study. In the present study, we detected a significant drop in IBV genome loads in the VC group compared to the NVC group at 3 and 7 dpi in lymphoid organs, such as BF and CT, in addition to other tissues, such as lung and kidney. Further, in the thymus (a primary lymphoid organ) and in tissues such as pancreas and duodenum, a significantly lower IBV genome load was recorded in the VC group compared to the NVC group at 3 dpi. Our results are indicative of a possible protection provided by maternal antibodies against IBV infection in primary (BF and thymus) and secondary (CT) lymphoid organs, in addition to other tissues, such as lung, kidney, pancreas, and duodenum. These findings are compatible with a recent study, which demonstrated that 1-day old chicks with higher maternal antibody levels had reduced IBV genome loads, and demonstrated early viral clearance in trachea and kidney when challenged with the Q1 IBV strain [26].

To further confirm the replication of IBV in lymphoid organs and the impact of the presence of maternal antibodies against IBV replication in lymphoid organs, immunohistochemistry technique was performed in order to demonstrate the expression of IBV nucleoproteins in infected tissues. Similar to the IBV genome loads, a remarkable amount of IBV nucleoprotein was detected in the lymphoid organs BF and CT at 3 and 7 dpi. In both BF and CT, at 3 dpi IBV nucleoprotein was detected in all the birds (100%) of the NVC group while only 87% of the birds (7/8 birds) were positive in the VC group. Hence, it could be suggested that the presence of maternal antibodies in the VC group provides a certain extent of protection against IBV replication in BF and CT at 3 dpi. In addition, in the thymus and spleen, none of the birds in the VC group were positive for IBV nucleoprotein, while 12.5% (1/8 birds) and 25% (2/8 birds) of the birds in the NVC group were recorded as IBV nucleoprotein positive in the thymus and spleen, respectively. Moreover, IBV nucleoproteins were detected in the trachea of both the VC and the NVC groups at 3 and 7 dpi. The expression of IBV nucleoproteins in lungs were detected at both 3 and 7 dpi in the NVC group, whilst it was detected only at 3 dpi in the VC group. The absence of IBV nucleoprotein and, thus, the absence of IBV replication in the thymus and spleen of the VC group could be due to the presence of maternal antibodies in the VC group. There are no previous studies in the literature that report on the impact of maternal antibodies against IBV replication in lymphoid organs by analyzing the presence of IBV nucleoprotein. Hence, these results provide valuable primary information for the formulation of future studies to determine the effect of a maternal antibodies against IBV replication in primary and secondary lymphoid organs. However, further quantitative studies are necessary to confirm that the presence of maternal antibodies negatively impact IBV replication in primary (BF and thymus) and secondary (CT and spleen) lymphoid organs as well as the potential impact on the cellular immune system. Furthermore, although we detected IBV genome in all of the birds in the primary (BF and thymus) and secondary (CT and spleen) lymphoid organs at both 3 and 7 dpi, not all of the birds were positive for IBV nucleoprotein in these tissues. The discrepancy in the detection of IBV genome load and IBV nucleoprotein in the primary and secondary lymphoid organs is not surprising, as qPCR detects both replicating and non-replicating IBV while the immunohistochemistry technique detects only IBV antigen expressed by the replicating virus [27]. Moreover, since qPCR is a much more sensitive technique when compared to immunohistochemistry technique, some tissues positive for IBV viral genome could be negative for IBV nucleoprotein.

There is limited literature reporting the presence of IBV-induced gross and microscopic lesions in primary (BF and thymus) and secondary (CT and spleen) lymphoid organs. IBV induced microscopic lesions have been previously described in BF [9,12,13] and spleen [12]. In the current study, microscopic lesions were observed in lymphoid organs BF and CT in addition to other tissues, such as trachea, lung, kidney, pancreas, and duodenum. In BF, kidney, and pancreas, the lesion score in the VC group was significantly lower than that in the NVC group at both 3 and 7 dpi. Further, the lesion score in CT and trachea was observed to be significantly lower in the VC group than that in the NVC group at 3 dpi. This reduction in IBV induced lesion scores in BF and CT in the VC group could be due to the protection provided by maternal antibodies in the VC group against IBV infection. Furthermore, IBV-induced lesions were not observed in the primary lymphoid organ thymus and the secondary lymphoid organ spleen at 3 and 7 dpi in both the VC and the NVC groups. This observation is congruent with a previous study where no IBV-induced histopathological lesions were observed in the thymus and spleen of chicks obtained from vaccinated hens [20] post-challenge with IBV. This study further reported that histopathological lesions were absent in BF of IBV-challenged chicks, which is contrary to the findings of our study [20]. This discrepancy in the observation of histopathological lesions in BF could be due to a number of factors, such as the difference in the breed of chicken, the strain of the IBV, and the IBV infection dose used in the two studies.

One limitation of this study is that it does not include the viral tropism and pathological damage in HG due to IBV DMV/1639 challenge in the birds. In future, studies assessing the viral tropism and the pathogenicity of the IBV DMV/1639 strain in HG could be performed. Further studies could be performed in order to determine and compare the cross protection provided by homologous and heterologous vaccine strains. It would be of great importance to determine and compare the immunological protective effect of DMV/1639 inactivated vaccine with Mass and Mass and Conn vaccines through cross-challenge experiments in adult hens and their progenies due to the protective effect of maternal antibodies. In addition, a study comparing the tissue tropism and pathogenicity between DMV/1639 strain to Mass and Conn strains could also be undertaken in the future.

In summary, we observed that the IBV antibody titer in serum was significantly higher in chicks from vaccinated hens compared to chicks from non-vaccinated hens, and, correspondingly, that the viral shedding in OP and CL swabs was significantly lower in chicks from vaccinated hens (VC) compared to chicks from non-vaccinated hens (NVC). In addition, we found that the IBV genome loads, and the histopathological lesions were significantly lower in chicks from the VC group compared to chicks from the NVC group in BF and CT. Although we still need further studies to determine a direct correlation between maternal antibody and its impact on IBV infection in lymphoid organs, our study provides preliminary data to suggest that the presence of maternal antibodies in chicks does provide some degree of protection against IBV infection in BF and CT up to 7 days post hatching.

## Figures and Tables

**Table 1:** The lesion scoring criteria for different tissues in infected birds.

Organ	Scoring Criteria
Trachea	Loss of cilia Epithelial cell degeneration and necrosis Mononuclear cell aggregation in lamina propria Hyperplasia of lining epithelium Loss of mucosa
Lung	Bronchitis Focal lymphoplasmocytic infiltration Diffuse lymphoplasmocytic infiltration Parabronchitis
Kidney	Tubular necrosis Focal lymphoplasmocytic infiltration Diffuse lymphoplasmocytic infiltration Focal lymphoid follicle aggregation Gout
BF	Depletion of lymphoid follicles Epithelial lining hyperplasia with squamous cell metaplasia Epithelial lining degeneration and necrosis
Thymus	Depletion of the cortical lymphocyte density or relative thickness to the medulla Thymus hemorrhage
Spleen	Hyperplasia or hypertrophy in the ellipsoids Lymphoidal apoptosis or necrosis Sinusoidal congestion
CT	Lymphoepithelial degeneration and necrosis Subepithelial zone inflammatory cell infiltration Lymphoid apoptosis and necrosis in germinal centers
Pancreas	Acinar cell necrosis Lymphoid infiltration Ductal dilation with inflammatory cell infiltration
Duodenum	Epithelial degeneration and necrosis Lymphoid infiltration in the lamina propria Submucosal lymphoid cell infiltration

**Table 2:** Expression of IBV nucleoprotein in affected tissues at 3 dpi.

	Trachea	Lung	Kidney	BF	CT	Spleen	Thymus	Duodenum	Pancreas
NVNC	0/8	0/8	0/8	0/8	0/8	0/8	0/8	0/8	0/8
VC	5/8	2/8	8/8	7/8	7/8	0/8	0/8	1/8	0/8
NVC	6/8	2/8	8/8	8/8	8/8	3/8	0/8	3/8	1/8
VNC	0/8	0/8	0/8	0/8	0/8	0/8	0/8	0/8	0/8

**Table 3:** Expression of IBV nucleoprotein in affected tissues at 7 dpi.

	Trachea	Lung	Kidney	BF	CT	Spleen	Thymus	Duodenum	Pancreas
NVNC	0/8	0/8	0/8	0/8	0/8	0/8	0/8	0/8	0/8
VC	7/8	0/8	7/8	8/8	8/8	0/8	0/8	1/8	0/8
NVC	8/8	5/8	7/8	8/8	8/8	2/8	1/8	3/8	0/8
VNC	0/8	0/8	0/8	0/8	0/8	0/8	0/8	0/8	0/8

## Data Availability

The datasets used and/or analyzed within the frame of the study can be provided by the corresponding author upon reasonable request.
